# Fluoroscopy Is Essential in Retrograde Intrarenal Surgery

**DOI:** 10.1155/2023/8896681

**Published:** 2023-11-29

**Authors:** Mehmet Yoldas, Tuba Kuvvet Yoldas

**Affiliations:** ^1^Tepecik Training and Research Hospital Clinic of Urology, Izmir, Türkiye; ^2^Tepecik Training and Research Hospital Clinic of Anesthesiology and Reanimation, Izmir, Türkiye

## Abstract

**Objective:**

This study aimed to investigate the necessity of using fluoroscopy in retrograde intrarenal surgery (RIRS). *Material and Methods*. A total of 612 patients who underwent RIRS for kidney stones were evaluated and divided into two groups. Group 1 routinely underwent the operation with fluoroscopy due to opaque stones (*n*: 504). In group 2, the procedure was performed without fluoroscopy because of nonopaque stones (*n*: 108). Both groups were assessed for stone size, location, and number. Success and complication rates were compared between the two groups.

**Conclusion:**

This study was designed with the thought of not using fluoroscopy in RIRS patients with nonopaque stones and having the same stone-free rates in opaque stones. In the statistical analysis, there was no difference between the groups with and without scope for stone side, size, localization, and number; likewise, the complication rates developed in the comparison of both groups, stone-free rates, and hospital stay were the same. *Discussion*. Advances in the calibration of instruments, the development of optical systems, and improvements in imaging system resolution have gradually reduced the need for fluoroscopy in RIRS. This study provides further evidence that fluoroscopy is unnecessary in RIRS procedures, thereby eliminating unnecessary radiation exposure.

## 1. Introduction

Stone disease affects 1–20% of the population, with a worldwide prevalence of 15–20% in regions like the stone belt, where Turkey is located [1]. In developed countries, the incidence surpasses 10%, and recent studies have indicated a 37% increase in some regions [2].

The incidence of stones is influenced by geographical, climatic, ethnic, dietary, and genetic factors [3].

With the evolution of minimally invasive techniques, stone surgeries have undergone significant transformations, predominantly adopting endoscopic approaches. As a result, the utilization of endoscopic instruments and fluoroscopy in these procedures has risen, leading to increased radiation exposure for both medical personnel and patients.

In tandem with these advancements, certain studies have demonstrated the feasibility of applying retrograde intrarenal surgery (RIRS) for stones larger than 2 cm [4, 5]. The routine use of fluoroscopy during RIRS is essential for various purposes, including defining renal calyx and ureter anatomy through retrograde pyelography, assessing the location of the ureteral access sheath, gaining access to stones, confirming stone-free status, and verifying the position of the stent and guide wire placement [6]. While minimally invasive procedures are beneficial for patients and staff, the need for radiation-free alternatives with comparable success rates has become imperative.

Nevertheless, guidelines maintain that fluoroscopy remains accessible during RIRS, providing an additional layer of assurance for surgeons and contributing to the prevention of complications.

## 2. Material and Methods

The study evaluated data from kidney stone patients who underwent retrograde intrarenal surgery (RIRS) between the years 2015 and 2022.

Patients with missing data, additional procedures with RIRS (PNC and so on), anomalies such as pelvic kidney collecting system multiplicity, patients with extracorporeal shock wave lithotripsy (ESWL) before the procedure, and patients who had a double J stent before the procedure were excluded from the study.

A total of 612 patients were included in the study.

This study received approval from the Local Ethics Committee of Tepecik Training and Research Hospital with approval number: 2021/12-12 dated 15.12.2021.

Patients were evaluated with computed tomography (CT) preoperatively, and full urinary analysis, brief biochemistry including urea, Cr, and hemogram, and urine culture were taken. RIRS procedure was performed by a single experienced surgeon or under the supervision of an expert at the Urology Clinic. Since nonopaque stones can be seen on CT, they can be controlled with CT. CT was performed in the 4th week after the procedure, and small residual stones of 4 mm or less were considered stone-free. The average follow-up period for the patients was 48.5 (12–84) months.

The patients were divided into 2 groups: group 1 (*n*: 504) is patients with opaque stones and group 2 (*n*: 108) is patients who did not use fluoroscopy and whose stones were not opaque. Demographic data of both groups were statistically compared for stone data and developing complications.

### 2.1. RIRS Procedure

Before the RIRS procedure, collecting system calibration was evaluated with a conventional ureterorenoscope (URS) and guide wire was placed. The ureteral accessory sheath was placed on the guide wire up to the level of the ureteropelvic junction. The sensor guide was removed, and the flexible URS ureteral accessory was inserted into the renal pelvis through the sheath. The scope was not used in the group whose stone was not opaque.

### 2.2. Statistical Analysis

In this study, statistical analyses were performed using the SPSS (Version 22.0; SPSS Inc., Chicago, IL, USA) package program. Descriptive statistics were presented as mean ± standard deviation for normally distributed continuous data, median (min-max) for nonnormally distributed continuous data, and numbers and percentages (%) for categorical data. Normality distribution was analyzed by the Kolmogorov–Smirnov test. In the comparison of numerical variables between two independent groups, *t*-test for normally distributed data was used for independent groups (student's *t*-test), and Mann–Whitney *U* test was used for data not normally distributed. Relations between Categorical variables, depending on the number of data in the crosstab cells tested with the Chi-square (Chisquare) or Fisher exact test. The statistical significance level was evaluated as *P* < 0.05.

## 3. Results

A total of 612 patients were analyzed, 504 patients in group 1 and 108 patients in group 2.

The mean age of the 1st group was 43.11 (19–74), and the mean age of the 2nd group was 41.18 (18–71). 312 (61%) men and 192 (29%) women are in group 1, and 76 (71%) men and 32 (29%) women are in group 2.

The mean stone sizes were 11.57 mm^2^ in group 1 and 11.43 mm^2^ in the other group. Both groups were equally distributed for age, sex, and stone size, respectively (P: 0.45 : 0.18 : 0.25).

312 patients had right kidney stones and 300 patients had left kidney stones. There was no difference between the two groups for the side of the stone (P: 0.26).

139 (27.5%) patients in group 1 and 23 ( 21.2%) in group 2had sinle and lower pole stones which the most difficult to access (P:0.13)

376 (%74.4) patients in group 1 and 78 (%70.2) patients in group 2 had a single stone (*P*: 0.10).

The mean number of patients in group 1 was 1047 and 684 out of group 2.

The preoperative data of the patients are given in Table 1.

The operation times were 52.35 (±13.16) minutes in group 1 and 50.58 (±12.56) minutes in group 2, and there was no significant difference between the two groups (*P*: 0.28).

Postoperative hospital stay is 1.16(±0.24) days in group 1 and 1.22(±0.25)days in group 2, respectivly (P:0.11).

, 27 (%5.3) patients in group 1 and 6(%5.5) patients in group 2 had postoperative complications. The majority of these complications were grade 1 and 2.

No blood product was transfused in any patient. After lithotripsy, DJS was inserted in 110 patients in group 1 and 26 patients in group 2. DJ stent may be displaced. The location of the DJS placed on the first postoperative day was checked with direct urinary system radiography. DJS migration was not observed in any of our patients. If it had been seen, early CT could have been performed and DJS could have been reinserted, including in non-opaque stones.

Fever developed in 4 patients after the procedure, and all patients recovered with outpatient antibiotic therapy.

Grade 3 A injuries are complications that can be managed without the need for general anesthesia and were observed in 6 patients in group 1 and 2 patients in group 2. Retroperitoneal collection developed in 2 patients in group 1, and procutaneous drainage was performed. Low-grade ureteral mucosal injury developed in 2 patients in group 1 and 1 patient in group 2, DJS or nephrostomy was placed in 2 patients in group 1 due to renal colic, and nephrostomy was placed in only 1 patient in group 2 due to renal colic.

Grade 3 B injury was observed in 2 patients in group 1: 1 of them was reoperated under general anesthesia due to the accumulation of stones in the distal ureter and other one due to the piece of guide wire remained in proximal ureter.

None of them had avulsion.

One patient in group 1 and 1 patient in group 2 due to postoperative sepsis were treated in the intensive care unit.

One patient died due to sepsis in group 1.

Operative complications and stone-free data of the patients are given in Table 2.

## 4. Discussion

Stone disease is widespread in urology practice, and the majority of this is kidney stones. Advancements in kidney stone surgery have led to increasingly noninvasive procedures. These advancements include reduced endoscope calibration, improved deflection mechanisms, enhanced optical resolution, and the evolution of laser technology. These developments have made minimally invasive methods more popular for treating kidney stones. However, with the growth of minimally invasive techniques, fluoroscopy usage has also increased. Fluoroscopy is utilized in RIRS procedures for placing a ureteral access sheath (UAS), stone navigation, and catheterizing the ureter to confirm catheter placement [7, 8]. Despite its advantages, the use of fluoroscopy exposes both patients and staff to radiation.

Stage 1-2 complications: RIRS complications are seen in 3–5% in some studies, with the majority of these complications falling within grades 1 and 2 [9]. In this study, in accordance with the literature, grade 1 and 2 complications were found to be predominantly higher, and general complications were found to be 27 (5.3%) in group 1 and 6 (5.5%) in group 2. Our rates were found to be higher than the literature because we are a high-volume hospital.

Urinoma and hematoma, as reported in the literature, are more likely to occur in elderly patients with hydronephrosis and infected stones [9, 10]. In our study, only 2 patients (0.4%) developed urinoma. Interestingly, in line with the existing literature, two of our patients were young and not on anticoagulants. It is possible that the presence of thin stones within the calyx parenchyma contributed to this complication. Percutaneous drainage and simultaneous DJ stent placement were performed in two patients. No urinoma or hematoma was observed in any of the patients in group 2.

### 4.1. Nephrostomy and DJS Insertion

Unresolved blood clots can lead to ureteral obstruction and renal colic. Clot formation and renal colic have been reported in 1–3% of cases after RIRS, often caused by minimal bleeding during stone fragmentation [11]. In our study, percutaneous nephrostomy and DJ stent insertion were required for 2 patients in group 1 and 1 patient in group 2 due to clot-related colic. All patients had multiple stones, with longer-than-average surgical durations. The drainage catheters were removed after 2 weeks.

UAS offers several advantages, including improved access to the collecting system, the ability for multiple accesses, reduced intrarenal pressure, enhanced drainage, protection of the area, and prevention of dilatation. Consequently, UAS has gained popularity worldwide. However, concerns arose about the potential for ureteral injuries associated with UAS use.

Bozkurt et al. conducted RIRS procedures on 39 patients without employing UAS. Stone-free rates in this study were found to be 84.6%. Notably, the success rates remained consistent in RIRS procedures conducted without UAS, while we argue that complication rates significantly decreased [11]. In our study, we observed mild ureteral mucosal damage in 8 patients (1.7%) in group 1 and 1 patient (2%) in group 2. All patients had the smallest UAS diameter of 9.5 Fr, and they were managed with a DJ stent. Several studies have investigated the potential for long-term ureteral strictures or short-term ureteral injuries associated with UAS use. On average, patients were followed up for 1 year, and ureteral stenosis occurred in only 0.5–1% of cases [12]. As a result of this study; it has been suggested that the application of UAS during RIRS reduces the operation time and costs and causes a decrease in morbidity, and it is recommended to be used routinely [13]. In our study, we aimed to prevent possible complications by using 9.5 Fr (CookMedical, Bloomington, USA), which is the smallest diameter through which the flex renoscope can pass. We did not detect long-term urethral structure in any of our patients.

Steinstrasse is a complication that necessitates reoperation under general anesthesia, and its incidence is estimated to be approximately 0.5–1%, with an increased risk associated with larger stone sizes [13]. Stone sizes exceeding 4 cm and the presence of larger stone fragments (1-2 mm) are known risk factors for steinstrasse formation. In our study, in line with previous literature, one patient (0.2%) developed a ureteral obstruction in the distal ureter, and the stones were subsequently managed with URSL. Notably, this complication was observed in a patient with a stone size of 3.2 cm, and it did not occur in the other group.

A foreign body refers to the breakage of stents used during the procedure, with some fragments remaining in the collecting system. Zisman et al. observed a dramatic decrease in fracture resistance in stents with fractures compared to those removed spontaneously [14]. Foreign bodies were extracted during a rerising procedure one month later. In some economically challenged countries, materials are sometimes used beyond their intended lifespan and are of lower quality due to cost considerations.

In our study, a patient in group 1 experienced the breakage of the hydrophilic part of the guide wire in the renal pelvis. This was detected during a postoperative CT scan at 4 weeks and was subsequently removed through reoperation. The patient had multiple stones, and the limited field of view caused by hematuria during the operation may have contributed to this complication.

### 4.2. Intensive Care Follow-Up and Death

Large-scale studies have reported a mortality rate of approximately 1 in 2000 patients, with the most common causes of death being cardiac issues, pulmonary complications, and sepsis [15]. In our study, two patients required intensive care unit monitoring due to sepsis, and unfortunately, one patient passed away due to sepsis and multiorgan failure. The deceased patient was 75-year-old with severe chronic obstructive pulmonary disease and diabetes. They had a single stone of approximately 1.5 cm^2^ in the renal pelvis, which was associated with hydronephrosis.

The stone-free rate in patients undergoing RIRS due to kidney stones varies between 70 and 90%. The success stone and its aggregate vary according to the characteristics of the system. Regarding the stone, its number and size are the main factors affecting its success, and the success rate decreases in cases of more than 2 cm and lower calyx stones [16, 17]. In our study, the RIRS procedure was performed with 82% stone-free status in group 1 and 86% in group 2, which is lower than the literature. This is because we are tertiary centers.

URS without fluoroscopy was first performed in the literature on distal ureteral stones by Mandhani et al. [18]. In another study examining proximal and distal ureteral stones, it was reported that fluoroscopy was needed in a patient group of 7.5% [19]. In a randomized controlled study conducted in recent years, no difference was found between stone-free and complications of URS accompanied and fluoroscopy-guided. Our study included patients who underwent RIRS operation for kidney stones, and both groups we compared had similar complications and success rates.

The limitations of our study can be counted as being retrospective, not including pregnant patients with some ureteral and/or kidney anomalies.

## 5. Conclusion

As a result, we have shown that the instruments used in RIRS can be used in uncomplicated patients with the opportunity provided by improved visualization systems, with the same complication and success rates without using fluoroscopy in experienced hands.

## Figures and Tables

**Table 1:** Preoperative data of the patients.

	Group 1 (504)	Group 2 (108)	*P*
Age	43.11 ± 3.4	41.18 ± 3.7	0.45
Sex			
Women	192 (29%)	32 (29%)	0.18
Men	312 (61%)	76 (71%)	
Stone size (mm^2^)	11.57 ± 2.6	11.43 ± 2.8	0.25
Laterality			
Right	254 (%51)	58 (%57)	0.26
Left	250 (%49)	50 (%43)	
Stone position			0.10
Lower pole	139 (%27.5)	28 (%25.9)	0.13
Middle pole	33 (%6.5)	18 (%7.4)	0.09
Upper pole	26 (%5.1)	9 (%8.3)	0.07
Pelvis	222 (%44.0)	43 (%39.8)	0.13
More than one pole	84 (%16.6)	18 (%16.6)	0.10
Number of stones			
1	376 (%74.4)	78 (%72.2)	0.10
2	98 (%19.3)	26 (%24)	
3	18 (%3.3)	2 (%1.9)	
4	11 (%2.0)	2 (%1.9)	
Stone Hounsfield unit (HU)	1047	684	0.001

**Table 2:** Operative complications and stone-free data of the patients.

		Group 1	Group 2	*P* value
Operation time (min.)		52.35 (±13.16)	50.58 (±12.56)	0.28

Scopy time (sc.)		0	24.29	

Postoperative hospitalization (days)		1.16 (±0.24)	1.22 (±025)	0.11

Complications		27 (%5.3)	6 (%5.5)	0.17

Degree 1-2	Use of antiemetics, antipyretics, analgesics, and so on	14 (%2.7)	2 (%0.2)	0.19
	Fever	3 (%0.5)	1 (%0.9)	0.15

Degree 3a	Hematoma, ürinoma	2 (%0.4)	0	
	Low-grade ureteral injury	2 (%0.4)	1 (%0.9)	0.09
	Nephrostomy insertion	1 (%0.2)	1 (%0.9)	0.08
	Installing postoperative DJS	1 (%0.2)	0	1.00

Degree 3b	URS again (due to ureteral stone)	1 (%0.2)	0	1.00
	Foreign body in the ureter (guide wire)	1 (%0.2)	0	1.00

Degree 4	Intensive care follow-up due to sepsis	1 (%0.2)	1 (%0.9)	0.19

Degree 5	Ex	1 (%0.2)	0	

Stone-free rate (SFR)		413 (%82)	93 (%86)	0.12

Installing postoperative DJS		110 (%21)	26 (%24)	0.13

## Data Availability

.The data that support the findings of this study are available on request from the corresponding author.
